# Quantitative Ultrasonographic Assessment of Supraspinatus Insertion Tendon in Non-Lame Dogs (Thickness and Relative Echogenicity)

**DOI:** 10.3390/ani16132100

**Published:** 2026-07-07

**Authors:** Juan Antonio Camara-Serrano, Hernan Fominaya-Garcia, Concepcion Rojo-Salvador, Angela Garau-Camacho, Pilar Llorens-Pena, Jesus Rodriguez-Quiros

**Affiliations:** 1 Preclinical Therapeutics Core, University of California San Francisco, San Francisco, CA 94158, USA; juanantonio.camaraserrano@ucsf.edu; 2 Department of Animal Medicine and Surgery, School of Veterinary Medicine, Complutense University of Madrid, 28040 Madrid, Spain; hernanfominaya@gmail.com (H.F.-G.); agarau@ucm.es (A.G.-C.); pllorens@ucm.es (P.L.-P.); 3 AniCura VETSIA Veterinary Hospital, C/Galileo 3, Leganes, 28914 Madrid, Spain; 4 Department of Anatomy and Embryology, School of Veterinary Medicine, University Complutense of Madrid, 28040 Madrid, Spain; crojosal@ucm.es

**Keywords:** ultrasonography, quantitative imaging, echogenicity, supraspinatus tendon, shoulder, dog

## Abstract

The canine supraspinatus insertion tendon (SIT) is frequently involved in shoulder disorders; however, objective ultrasonographic reference data remain limited. This study aimed to describe the ultrasonographic appearance of the SIT in clinically non-lame dogs and to develop a reproducible protocol for the quantitative analysis of tendon thickness and relative echogenicity. Thirty-five clinically non-lame dogs (70 shoulders) underwent prospective B-mode ultrasonography. On ultrasonographic examination, the SIT appeared as a structure composed of two clearly differentiated regions: a proximal region, which was thinner and more hyperechoic, and a distal region, which was thicker, relatively more hypoechoic, and attached to the bone surface. These findings were consistent with previous descriptions of the SIT ultrasonographic appearance reported in the literature. Tendon thickness and mean echogenicity were evaluated in both regions. Mean echogenicity was quantified using region-of-interest analysis and pixel intensity histograms, with trapezius muscle echogenicity used for background normalisation. Paired statistical tests and Pearson correlation analyses were performed. No significant differences were identified between the left and right SIT measurements for either thickness or relative echogenicity (*p* > 0.05). The distal region was significantly thicker than the proximal region (*p* < 0.001). Relative echogenicity also differed significantly between regions: the proximal region was more hyperechoic and heterogeneous, whereas the distal region was more homogeneous and hypoechoic (*p* < 0.001). Body weight and height were positively associated with tendon thickness, whereas associations with mean echogenicity were reduced after normalisation to trapezius muscle echogenicity. In conclusion, the canine SIT comprises two ultrasonographically distinct regions that differ in thickness and echogenicity. The proposed protocol provides an objective and standardized method for the quantitative ultrasonographic evaluation of tendon structure in dogs. However, this study represents a preliminary assessment of the SIT ultrasonography, and a larger sample size is required to confirm the statistical results obtained.

## Simple Summary

Supraspinatus tendinopathy is a recognised cause of forelimb lameness in dogs. The condition predominantly affects large and active breeds, and its diagnosis relies primarily on ultrasonographic examination, which enables non-invasive assessment of tendon morphology and echogenicity. Despite its clinical relevance, standardised ultrasonographic protocols for the objective evaluation of the canine supraspinatus tendon remain lacking. The aim of this study was to describe the ultrasonographic appearance of the supraspinatus insertion tendon in clinically non-lame dogs and to develop a standardized quantitative protocol for the quantitative analysis of tendon echogenicity. The results indicate that the canine supraspinatus insertion tendon comprises two ultrasonographically distinct regions characterised by differences in thickness and echogenicity. The quantitative protocol described here provides a standardized quantitative method for the objective ultrasonographic evaluation of tendon structure. These findings suggest that quantitative ultrasonographic assessment may improve the objective evaluation of supraspinatus tendon morphology in dogs, and facilitate the early detection and monitoring of tendon pathology in clinical practice.

## 1. Introduction

The canine shoulder (glenohumeral) joint is a highly mobile articulation with limited osseous congruence; consequently, joint stability relies primarily on surrounding soft tissues, including the joint capsule, ligaments, and the musculotendinous structures of the rotator cuff [1]. Among these, the supraspinatus tendon plays a crucial role in joint stabilisation and is frequently involved in shoulder pathology [2,3,4,5,6,7,8,9,10]. Shoulder soft-tissue disorders account for approximately 50% of forelimb pathologies in dogs, and supraspinatus disorders represent nearly 20% of these conditions, predominantly affecting the SIT [6,8,9,10,11].

The principal pathologies affecting the SIT in both human and veterinary medicine include tendinitis, tendinosis, tendon tears, and calcification [12,13]. These conditions are commonly characterised by alterations in tendon morphology and echogenicity. In both species, the distal portion of the tendon near the insertion site is considered the most clinically relevant region because it is more frequently associated with degenerative changes, calcific tendinopathy, and chronic injury [8,12,13]. Reduced vascularisation near the tendon insertion has been proposed as one of the key factors contributing to the increased susceptibility of this region to degeneration and impaired healing in humans [14,15,16,17,18].

Although supraspinatus pathology has been extensively investigated in humans, information in veterinary species remains limited. In dogs, a retrospective study by Canapp et al. [8] demonstrated that the deep central fibres adjacent to the tendon insertion were the regions most associated with supraspinatus tendinopathy, mineralisation, fibre disorganisation, and pain-related lameness. Research in other species—such as horses, cats, or sheep—remains scarce and is mainly descriptive or experimental [19,20,21,22,23,24,25]. Consequently, comparisons with the human literature remain highly relevant due to the extensive characterisation of the supraspinatus tendon in human shoulder pathology.

Ultrasonography is widely used for the evaluation of musculoskeletal soft tissues because it is non-invasive, accessible, relatively inexpensive, and allows real-time dynamic assessment [26,27,28,29,30,31,32,33,34,35,36,37,38]. In both human and veterinary medicine, it is considered particularly suitable for tendon evaluation owing to its high sensitivity in detecting structural abnormalities and changes in tendon echogenicity [39,40,41,42,43,44,45,46,47,48,49,50,51,52,53,54,55,56,57,58,59,60,61,62,63,64,65,66]. In veterinary medicine, specifically, ultrasonography has become an increasingly important tool for the assessment of shoulder disorders, including supraspinatus pathology [26,27,31,32,34,35,36,37].

Despite these advantages, ultrasonography remains highly operator-dependent in human and veterinary medical fields, particularly regarding the subjective interpretation of tissue echogenicity [44,45]. In certain tendon disorders, echogenicity alterations may represent the only detectable abnormality; however, the absence of standardised quantitative protocols limits reproducibility and objective comparison. Recent studies in humans have therefore focused on quantitative echogenicity analysis as an indirect method for assessing tendon composition and collagen fibre organisation [46,48,52,54,55,57,59,63,64,65,66].

The present preliminary study aimed to describe the ultrasonographic appearance of the SIT in asymptomatic dogs and to develop a reproducible protocol for the quantitative assessment of tendon thickness and mean echogenicity. In addition, the relationship between ultrasonographic measurements and demographic characteristics of the examined dogs was evaluated to identify potential biological factors influencing the ultrasonographic appearance of the SIT.

## 2. Materials and Methods

### 2.1. Study Design

This prospective study included 35 non-lame dogs (22 males, 13 females) presented to the Clinical Veterinary Hospital of the Universidad Complutense de Madrid (Madrid, Spain) for reasons unrelated to the study. Recorded variables included sex, age, body weight, height, and body mass index (BMI), calculated as weight (kg)/height (m^2^).

The absence of lameness and shoulder pain was confirmed through complete orthopaedic examination and a detailed anamnesis performed by a veterinary clinician with more than 20 years of experience. The examination included palpation and assessment of shoulder hyperflexion, hyperextension, abduction, adduction, and rotational movements. Owners completed a questionnaire addressing previous forelimb pain or lameness during the preceding two years.

Both shoulders were examined in each dog, resulting in 70 evaluated shoulders. No sedation or anaesthesia was required. All procedures followed institutional guidelines for humane animal care, and written informed consent was obtained from all owners prior to inclusion.

All ultrasonographic examinations were performed by an experienced veterinary radiologist using a Mindray DP6600 Vet ultrasound system (Mindray Biomedical Electronics, Shenzhen, China) equipped with a 5–10 MHz linear probe (75L60EA). Acquisition parameters—including gain, depth, focus, frequency, dynamic range, and time-gain compensation—were individually adjusted to optimise tendon visualisation according to patient size and scanning conditions. This approach was intentionally selected to reflect routine clinical practice and to evaluate an image-analysis strategy capable of reducing the influence of acquisition variability on quantitative echogenicity assessment. No phantom-based calibration was performed.

### 2.2. Ultrasonographic Examination

The scanning protocol was adapted from Long and Nyland [27]. Dogs were positioned in lateral recumbency, the shoulder region was clipped, and ultrasound gel was applied. The examined forelimb was extended caudally. After palpation of the scapular spine, the transducer was initially positioned transversely over the spine to identify the supraspinatus muscle. The probe was then moved proximally within the supraspinatus fossa until the musculotendinous junction was identified and subsequently rotated 90° clockwise to obtain a longitudinal view of the supraspinatus insertion tendon (SIT).

Minor probe adjustments were performed to identify the point of maximal tendon thickness and minimise anisotropy artefacts. Images were then recorded for analysis. The same protocol was repeated for the contralateral shoulder.

Measurements in the present study were performed in longitudinal scanning planes because tendon evaluation was conducted following the European Society of Musculoskeletal Radiology guidelines for supraspinatus ultrasonography, which recommend a longitudinal imaging approach. Longitudinal imaging provides superior visualisation of the fibrillar architecture, facilitates reproducible identification of the supraspinatus footprint, minimizes anisotropy-related artifacts, and allows more reliable perpendicular calliper placement compared with transverse imaging.

### 2.3. Image Analysis

Images were digitally stored in JPEG format, anonymised, and analysed in a blinded manner by the same examiner using ImageJ software (version 1.54p; NIH, Bethesda, MD, USA). Although DICOM is considered the reference standard for medical imaging, JPEG images preserved sufficient information for the grayscale analyses performed in this study.

SIT thickness was measured in the longitudinal plane at proximal and distal tendon regions. The proximal measurement was obtained immediately before tendon widening, whereas the distal measurement corresponded to the point of maximal tendon thickness over the humeral insertion. Measurements were performed perpendicular to the tendon’s longitudinal axis.

Mean tendon echogenicity was assessed by manually placing regions of interest (ROIs) over the selected tendon areas. ROI size was adapted to anatomical variability. A fixed-size control ROI was positioned over the trapezius muscle to provide a background reference for grayscale intensity normalisation.

Quantitative echogenicity analysis included pixel intensity histograms, mean intensity, standard deviation, coefficient of variation, and intensity range. Relative echogenicity values were obtained by subtracting trapezius muscle mean intensity from tendon mean intensity values.

### 2.4. Statistical Analysis

Statistical analysis was performed using SigmaPlot^®^ software (version 15.0.0.13; Grafiti LLC. 405 Waverley St Palo Alto, CA 94301). Left and right shoulders, as well as proximal and distal tendon regions, were compared using paired *t*-tests. Statistical significance was set at *p* < 0.05 with a 95% confidence interval.

Associations between ultrasonographic measurements and biological variables were evaluated using Pearson correlation analysis. Sex-related differences were analysed using *t*-tests. Multiple linear regression analyses were performed to investigate the association between biological variables and ultrasound-derived SIT measurements. The dependent variables included mean proximal tendon thickness, mean distal tendon thickness, mean proximal relative echogenicity, and mean distal relative echogenicity.

Age, sex, body weight, height, and BMI were initially evaluated as potential explanatory variables. Multicollinearity among independent variables was assessed using variance inflation factors (VIFs). Due to substantial collinearity among body-size-related variables, body weight was retained as the primary indicator of body size, whereas height and BMI were excluded from the final models. Consequently, the final regression analyses included age, body weight, and sex as explanatory variables.

Sex was coded as a binary variable (male = 1, female = 0). Model assumptions were assessed through residual diagnostics, including evaluation of normality using the Shapiro–Wilk test and homoscedasticity using the Spearman rank correlation test. Statistical significance was established at *p* < 0.05.

Measurement repeatability and reproducibility were assessed using Gauge Repeatability and Reproducibility (Gauge R&R) analysis. Each ultrasonographic target was measured four consecutive times under identical analytical conditions. Reliability assessment included repeatability, reproducibility, coefficient of variation, and intraclass correlation coefficients (ICCs).

Normality of data distribution was assessed using Kolmogorov–Smirnov and/or Shapiro–Wilk tests. Parametric analyses were applied when normality and homogeneity of variances were confirmed; otherwise, the corresponding non-parametric tests were used.

## 3. Results

### 3.1. Population Description

Thirty-five clinically non-lame dogs were included in the study. The mean age was 12.23 years, mean body weight was 23.23 kg, and mean height was 46.16 cm. Descriptive statistics for the study population are presented in Table 1.

### 3.2. Ultrasonographic Appearance of the Tendon

The SIT was successfully visualised in all cases without technical difficulty. In the longitudinal view, the tendon originated within the supraspinatus muscle as converging hyperechoic fibres that progressively merged distally. Near the insertion site, the tendon became abruptly thicker, more hypoechoic, and more homogeneous, with reduced visibility of the internal fibrillar architecture.

**Figure 1:**
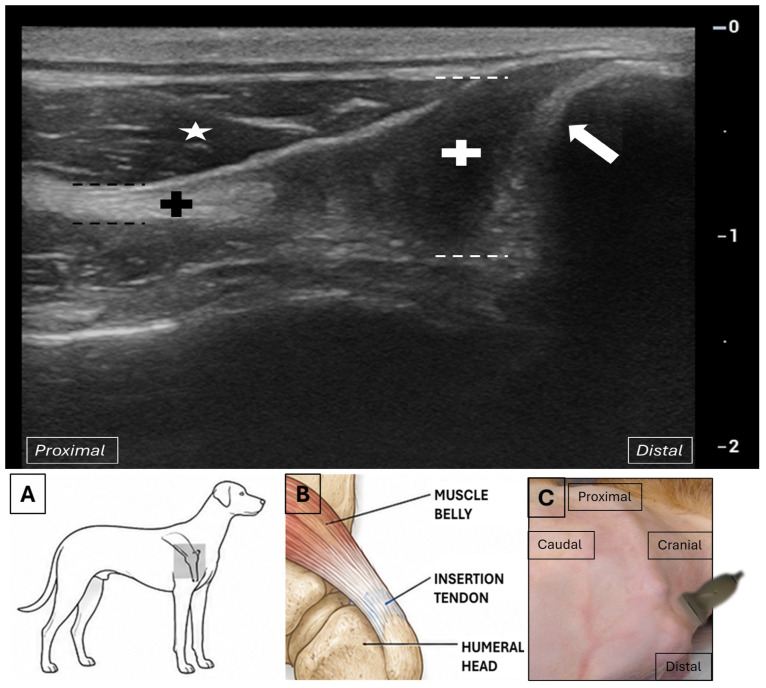
(**Top**): Representative longitudinal ultrasonographic image of the canine SIT showing tendon thickness measurements. The black cross indicates the proximal region of the SIT, while the white cross marks the distal region near the tendon insertion. Black dashed lines delimit tendon thickness in the proximal region, whereas white dashed lines indicate thickness measurement in the distal region. The white star marks the trapezius muscle used as a reference tissue for relative echogenicity analysis. The white arrow indicates the humeral surface at the tendon insertion site. The depth scale is shown in centimetres (cm). (**Bottom**): (**A**) Schematic representation of the canine shoulder location. (**B**) Schematic anatomy of the supraspinatus insertion tendon. (**C**) Probe positioning over the canine shoulder for longitudinal ultrasonographic examination with anatomical orientation marks.

The proximal tendon region appeared thinner, more hyperechoic, and more heterogeneous than the distal region, which attached broadly to the humeral surface. The tendon–bone interface was identified as a hyperechoic line with marked acoustic shadowing (Figure 1).

**Figure 2:**
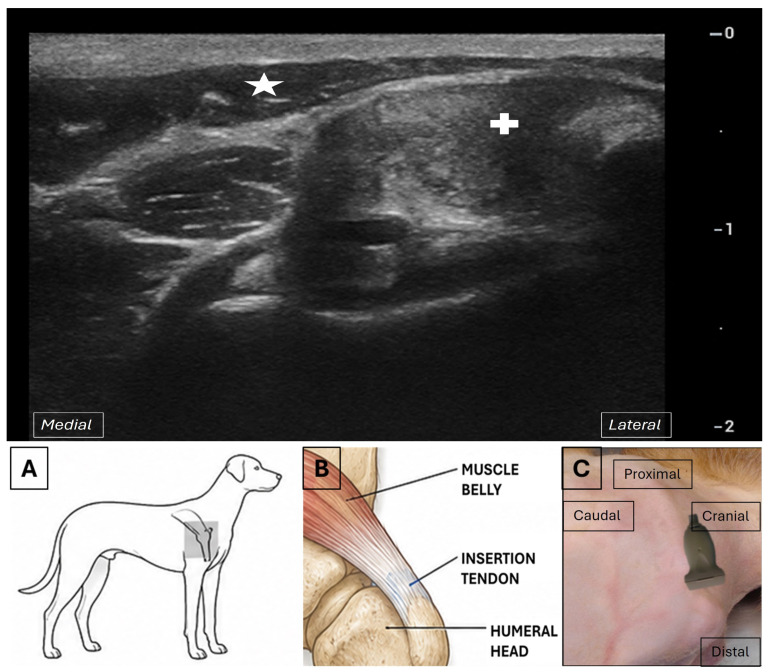
(**Top**): Representative transverse ultrasonographic image of the distal portion of the canine SIT. The white cross indicates the distal SIT, while the white star marks the trapezius muscle used as a reference tissue for echogenicity analysis. The proximal portion of the tendon is not included in this imaging plane; however, a complete cross-sectional visualisation of the distal SIT is obtained. The depth scale is shown in centimetres (cm). (**Bottom**): (**A**) Schematic representation of the canine shoulder location. (**B**) Schematic anatomy of the supraspinatus insertion tendon. (**C**) Probe positioning over the canine shoulder for transverse ultrasonographic examination with anatomical orientation marks.

In the transverse view, the SIT showed an oval shape and homogeneous echotexture. As observed in the longitudinal view, the distal region appeared thicker and more hypoechoic than the proximal portion (Figure 2).

### 3.3. SIT Thickness Measurements

Thickness measurements obtained in the longitudinal plane are summarised in Table 2. No significant differences were identified between the left and right shoulders in either tendon region (*p* > 0.05).

**Figure 3:**
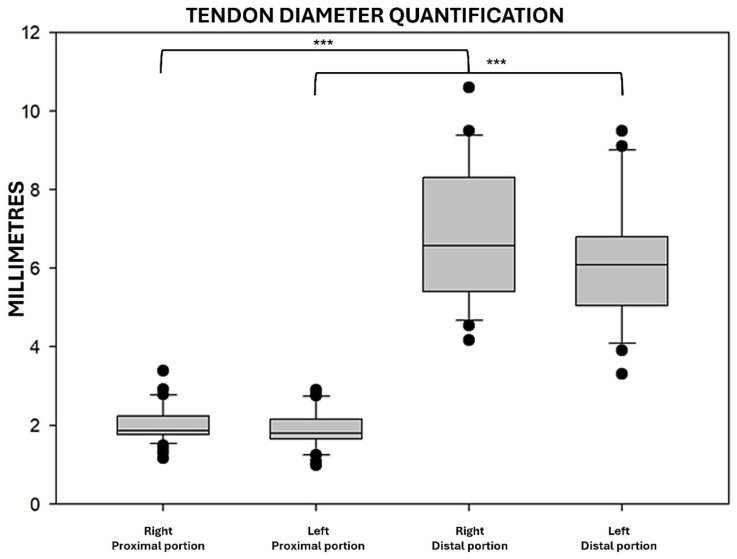
Boxplots showing thickness measurements of the proximal and distal regions of the canine SIT in the left and right shoulders. Boxes represent the interquartile range, the central line indicates the median, and whiskers represent the data range. Asterisks indicate significant differences between proximal and distal tendon regions in both shoulders (*** *p* < 0.05).

In contrast, significant differences were observed between the proximal and distal regions (*p* < 0.05), with the distal region being substantially thicker than the proximal region (mean difference of 4.46 millimetres) (Figure 3). This marked difference facilitated consistent ultrasonographic identification of both tendon regions.

### 3.4. Echogenicity Analysis

Mean echogenicity values for the SIT and trapezius muscle are shown in Table 3.

**Figure 4:**
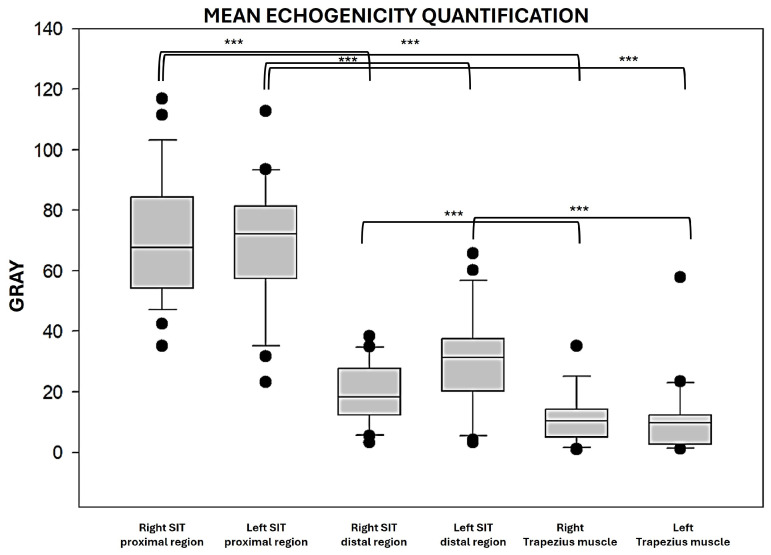
Boxplots show echogenicity values of the proximal and distal regions of the canine SIT and the trapezius muscle used as reference tissue in both shoulders. Echogenicity is expressed as grayscale intensity values. Boxes represent the interquartile range, the central line indicates the median, and whiskers represent the data range. Significant differences between structures are indicated by asterisks (*** *p* < 0.05).

Significant differences in mean echogenicity were identified between proximal and distal tendon regions, whereas no differences were observed between the left and right shoulders. The proximal region was more hyperechoic and heterogeneous, whereas the distal region appeared more homogeneous and hypoechoic (Figure 4).

After correction using trapezius muscle echogenicity, no significant differences were found between the left and right tendons (*p* > 0.05). Corrected relative echogenicity values are presented in Table 4.

**Figure 5:**
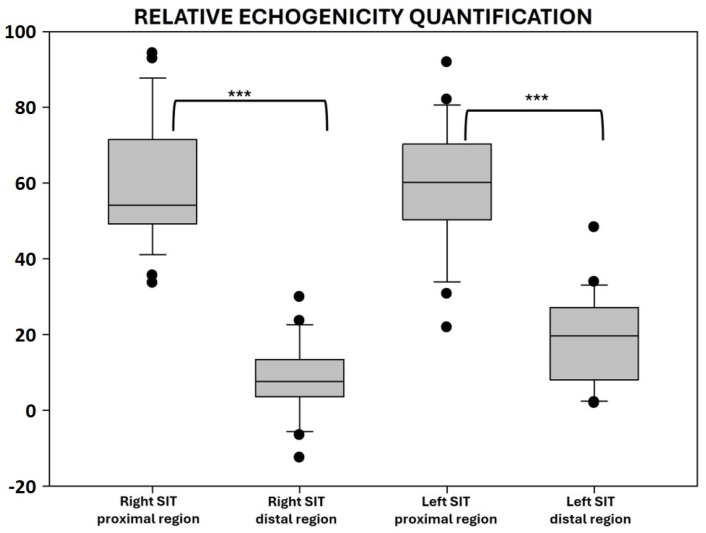
Boxplots showing corrected relative echogenicity values of the proximal and distal regions of the canine SIT in the left and right shoulders. Echogenicity values were normalised using trapezius muscle echogenicity as reference tissue and are expressed as ultrasound grayscale intensity values. Boxes represent the interquartile range, the central line indicates the median, whiskers represent the data range, and dots indicate outliers. Significant differences between proximal and distal tendon regions are indicated by asterisks (*** *p* < 0.001).

Comparisons between proximal and distal tendon regions remained significantly different after correction (*p* < 0.001), with higher relative echogenicity values in the proximal region (Figure 5).

**Figure 6:**
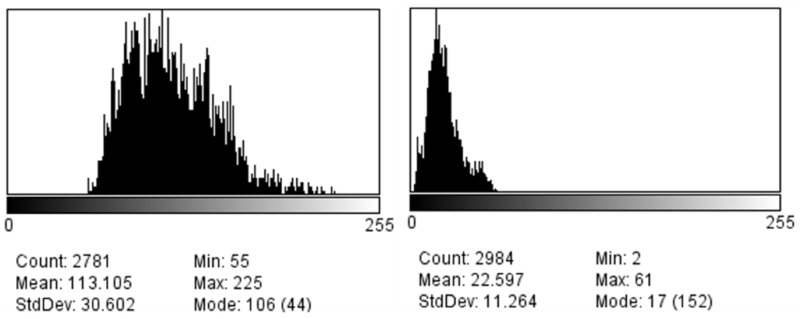
Representative grayscale intensity histograms of ultrasound images from the proximal (**left**) and distal (**right**) SIT regions. Compared with the distal region, the proximal region shows higher mean pixel intensity values and a wider distribution of grayscale intensities, while the distal region demonstrates greater homogeneity and a more compact distribution.

Representative histograms are shown in Figure 6. The proximal region demonstrated higher pixel intensity values and a broader distribution range, consistent with greater echogenic heterogeneity. In contrast, the distal region showed a narrower and more homogeneous intensity distribution.

### 3.5. Relationship Between SIT Measurements and Biological Variables

Pearson correlation analyses were performed to evaluate relationships between tendon measurements and biological variables. Because left and right shoulder measurements were not statistically independent, bilateral values were averaged for each dog prior to inferential analyses. Preliminary comparisons confirmed the absence of significant differences between shoulders, supporting this approach. The results of the Pearson correlation analysis are summarised in Table 5.

Sex was not significantly associated with any tendon parameter (*p* > 0.05). Age showed no relevant associations either. In contrast, body weight demonstrated significant positive correlations with tendon thickness in both the proximal and distal regions. Height also correlated positively with tendon thickness. These findings indicate that larger dogs tend to possess thicker supraspinatus tendons.

Body weight and BMI initially showed positive correlations with raw mean echogenicity values in certain regions; however, these associations disappeared after background correction using trapezius muscle echogenicity. This finding supports the implementation of background normalisation for the quantitative comparison of tendon echogenicity across different ultrasonographic images.

Multiple linear regression analyses were performed using sex, age, and body weight as independent variables. BMI and height were excluded due to multicollinearity and limited biological relevance. Body weight was identified as a significant independent predictor of tendon thickness, particularly in the distal region. Proximal tendon thickness showed a moderate association with weight (R^2^ = 0.237, *p* = 0.012), whereas distal tendon thickness demonstrated a stronger association (R^2^ = 0.558, *p* < 0.001). In contrast, echogenicity parameters showed weak and non-significant relationships with demographic variables.

### 3.6. Repeatability and Reproducibility Analysis

Gauge Repeatability and Reproducibility (Gauge R&R) analyses demonstrated high reliability of ultrasonographic measurements. Tendon thickness measurements showed low variability (CV ≈ 3.2%) and excellent reproducibility (ICC ≈ 0.97). Echogenicity measurements also demonstrated excellent reproducibility, with ICC values of approximately 0.992 and a mean coefficient of variation of approximately 2.84%.

In both analyses, measurement-related variability was substantially lower than the biological variability observed between animals, supporting the reliability and suitability of the proposed ultrasonographic protocol for objective quantitative tendon assessment.

## 4. Discussion

The present preliminary study provides a quantitative and standardised ultrasonographic evaluation of the SIT in clinically healthy dogs. The main findings include the identification of two distinct ultrasonographic tendon regions, the absence of significant bilateral differences, and the influence of body size on tendon dimensions. In addition, this study proposes a quantitative methodology for the assessment of mean and relative tendon echogenicity combined with image normalisation, representing a methodological advance over previous canine studies that relied mainly on qualitative or semi-quantitative ultrasonographic descriptions. However, due to the small sample size of this preliminary study, further research with larger sample sizes is required to validate the conclusions obtained herein.

### 4.1. Ultrasonographic Exploration and Image Analysis of the SIT

The ultrasonographic exploration protocol followed the methodology previously described in canines by Long and Nyland [27] and subsequently applied in later canine shoulder studies [26,28,29,30,31,32,34,35,36,37]. Although previous publications have described the ultrasonographic appearance of the canine SIT, most lacked standardised quantitative analysis protocols. In human medicine, tendon dimensions are commonly evaluated using tendon thickness measurements at predefined anatomical regions, whereas quantitative echogenicity analysis remains highly variable, with differences in ROI selection, image normalisation, and correction methods [39,40,41,42,43,44,45,46,47,48,49,50,51,52,53,54,55,56,57,58,59,60,61,62,63,64,65,66].

In the present study, variable ROIs were selected because tendon size varied considerably among dogs, making fixed ROIs potentially less representative of the true tendon structure. Mean echogenicity was analysed rather than total pixel count, as this approach better reflects clinical variability while minimizing sampling bias. Background correction was additionally implemented to mitigate the influence of acquisition-related variability on image intensity measurements. Although background normalisation is uncommon in musculoskeletal ultrasonography, similar correction methods are routinely applied in other human imaging modalities, such as PET [67]. Because ultrasonographic intensity can be affected by technical factors -including gain settings, coupling gel distribution, and tissue composition- normalisation may improve the reliability of inter-patient comparisons.

The trapezius muscle was selected as the reference structure because of its anatomical proximity and consistent visualisation during SIT examination. No significant differences in trapezius mean echogenicity were identified between sides, supporting its suitability as a normalisation reference. Nevertheless, the use of the trapezius muscle as a reference tissue should be considered a pragmatic normalization strategy based on its anatomical accessibility and apparent echogenic stability in the present study. Further investigations are warranted to validate its suitability as a reference tissue across different canine populations, imaging systems, and pathological conditions. Importantly, correction was applied only to mean intensity values, whereas parameters derived from pixel distribution, such as standard deviation and coefficient of variation, were not influenced by global image brightness.

### 4.2. Ultrasonographic Appearance and Quantitative Characteristics of the SIT

The ultrasonographic appearance observed in this study closely corresponded with previously published anatomical and ultrasonographic descriptions of the canine supraspinatus tendon [1,2,3,6,26,27,28,29,31,32,33,34,35,36,37]. The canine SIT was identified as a fibrillar structure that progressively broadened near its humeral insertion, forming a wide distal attachment area comparable to the “footprint” described in human medicine [14,15,16,17,18]. As the tendon approached the insertion site, echogenicity decreased and the tendon became wider and more homogeneous. These findings are consistent with previous cadaveric observations in canines reported by Lassaigne et al. [36], although tendon echogenicity in that study was assessed only qualitatively.

No significant differences were observed between the left and right shoulders, supporting the absence of functional limb dominance effects in dogs, unlike findings reported in human medicine [40,44,46,49,51,52]. Bilateral tendon evaluation remains clinically relevant because it allows intraindividual comparison and may facilitate identification of subtle pathological changes. The distal tendon region was significantly thicker than the proximal region, likely reflecting adaptation to the broad tendon–bone insertion interface and the fibrocartilaginous transition zone near the humeral attachment. This configuration may contribute to joint stability and efficient force transmission in the canine shoulder.

Quantitative evaluation of mean tendon echogenicity demonstrated clear regional differences between the proximal and distal SIT. The proximal region was more hyperechoic and heterogeneous, whereas the distal region appeared thicker, more homogeneous, and relatively hypoechoic. These differences likely reflect regional variations in collagen organisation, fibre orientation, and fibrocartilaginous composition near the insertion site.

Importantly, the correlations between tendon mean echogenicity and biological variables changed substantially after image normalisation, highlighting the influence of acquisition-related brightness variability on quantitative ultrasonographic analyses. These findings support the potential usefulness of background normalisation when comparing echogenicity values among animals examined under variable clinical imaging conditions.

The observed correlations between trapezius muscle echogenicity and body weight suggest that tissue composition may influence overall ultrasonographic brightness. Heavier or obese animals may present increased tissue hyperechogenicity because of intramuscular fat deposition or reduced muscular activity, as previously described in human studies [52,54,55,57,58,63,64,65]. Similar relationships were initially identified between tendon mean echogenicity and body weight; however, these correlations disappeared after normalisation, suggesting that certain echogenicity differences may reflect global image brightness rather than intrinsic tendon composition. Consequently, normalisation may improve the interpretability of quantitative tendon echogenicity measurements in heterogeneous patient populations.

An additional consideration is the age distribution of the study population, which consisted predominantly of older dogs (mean age 12.2 years). Consequently, the ultrasonographic characteristics described in the present study may be more representative of clinically asymptomatic aging tendons than of the general canine population. Although all enrolled dogs were free of clinical signs of shoulder disease, age-related structural tendon changes cannot be completely excluded. Therefore, the reference values reported herein should be interpreted as representative of clinically asymptomatic older dogs rather than universally applicable to dogs of all ages.

### 4.3. Relationships Between Tendon Characteristics and Biological Variables

Sex, age, and morphometric variables were analysed to provide biological context for the ultrasonographic findings. In contrast to human studies reporting increased rotator cuff pathology prevalence in older women [40,51,58,63,64,65], no sex-related differences in tendon thickness or echogenicity were observed in the present canine population. Age demonstrated a mild positive association with proximal tendon thickness, which could reflect early asymptomatic degenerative changes; however, no associated echogenicity alterations were identified. Because the study population consisted of clinically healthy dogs, larger studies including broader age distributions may be necessary to better characterise age-related tendon changes.

Body size demonstrated the strongest association with tendon morphology. Larger and heavier dogs presented thicker tendons, particularly in the distal region, supporting the biomechanical concept that tendon dimensions scale according to loading demands and body mass. These findings are consistent with previous canine studies [36] and with general musculoskeletal scaling principles observed in other anatomical structures. In contrast, tendon echogenicity showed weak or inconsistent relationships with morphometric variables after image normalisation, suggesting that tendon composition may be relatively independent of body size under normal physiological conditions.

BMI and height were initially explored as potential covariates but were ultimately excluded from the final regression analyses because they demonstrated substantial collinearity with body weight and did not improve model performance. Furthermore, BMI has limited biological validation in dogs because of marked breed-related differences in body conformation. Body weight was therefore retained as the most clinically relevant and statistically robust morphometric variable for the final analyses. Overall, the present findings suggest that tendon thickness is influenced predominantly by body size and mechanical loading, whereas tendon relative echogenicity appears less dependent on anthropometric variation in clinically non-lame dogs.

### 4.4. Clinical Relevance and Future Directions

The quantitative protocol proposed in this study may improve the objectivity and standardization of canine tendon ultrasonography and could facilitate the early detection of subtle or subclinical tendon abnormalities. Establishing reference values in clinically healthy dogs may also support future comparative studies in dogs with supraspinatus tendinopathy. In addition, quantitative assessment of tendon echogenicity may enable longitudinal monitoring of tendon healing and degeneration, providing an objective method to evaluate disease progression and response to treatment. This may be particularly relevant in conjunction with ultrasound-guided therapeutic interventions, which are increasingly employed for the local administration of regenerative and other targeted treatments in canine tendon disorders [68,69].

### 4.5. Study Limitations

Several limitations should be acknowledged in this exploratory study. The sample size was relatively small and included only clinically non-lame dogs, limiting the establishment of definitive reference values and the evaluation of weaker biological associations. In addition, no histopathological correlation was available because of the in vivo nature of the study.

Another limitation of the present study is the absence of a formal inter-observer reliability assessment. All ultrasound examinations and image measurements were performed by a single examiner. Consequently, although intra-observer repeatability was evaluated, the reproducibility of the proposed protocol across different operators could not be determined. Future studies involving multiple observers with different levels of experience are warranted to assess the inter-observer reliability and generalizability of the described ultrasonographic methodology.

Quantitative measurements may also be influenced by ROI selection, tendon anisotropy, and the use of the trapezius muscle as a reference tissue for echogenicity normalisation. Although efforts were made to standardise image acquisition and minimise these effects, these factors may influence quantitative ultrasonographic analysis. The use of JPEG image format could affect quantitative results due to the potential loss of image information during file compression; however, data loss in grayscale images is considerably lower than in coloured pictures. The lack of fixed scanning settings and a calibration phantom represents another limitation in terms of technical homogeneity of the scans.

Furthermore, while cadaveric tendon measurements are useful for anatomical standardisation and comparative studies, they may not fully reproduce in vivo tendon characteristics because post-mortem changes can alter tendon morphology and ultrasonographic appearance. Therefore, further studies including pathological tendons and larger study populations are required to validate the clinical applicability of quantitative SIT ultrasonography.

## 5. Conclusions

This preliminary study describes the ultrasonographic characteristics of the SIT in non-lame dogs and presents a standardized protocol for quantitative echogenicity analysis. Bilateral comparisons revealed no significant differences in tendon thickness or mean echogenicity, supporting the use of the contralateral limb as a reliable clinical control. Two distinct regions were identified within the SIT: the proximal region is thinner, heterogeneous, and hyperechoic, whereas the distal region is thicker, homogeneous, and hypoechoic, likely reflecting regional variations in tendon organisation and insertional architecture. Tendon thickness correlated positively with body weight and height, whereas relative echogenicity showed weaker associations with anthropometric variables. The proposed protocol provides a standardized framework for objective tendon assessment and may be applicable to other musculoskeletal structures. Further studies involving larger populations and pathological cases are required to establish definitive reference ranges and to evaluate the diagnostic value of quantitative echogenicity in clinical practice.

## Figures and Tables

**Table 1:** Biological characteristics of the canine study population (*n* = 35).

	Mean	SD	Coefficient of Variation	Range
**Age (years)**	12.23	2.25	0.18	9
**Weight (kg)**	23.23	14.70	0.63	55
**Height (cm)**	46.16	14.96	0.32	53
**BMI (kg/m^2^)**	107.55	48.41	0.45	239
**Breed (*n*)**	Old English Sheepdog, Boxer, Spanish Mastiff, Catalan Sheepdog, Briard (Brie Sheperd), Scottish Terrier, Dachshund, West Highland White Terrier: 1 Husky, Pointer, Shih Tzu: 2 Fox terrier, Retriever: 3 Poodle: 4 German Shepherd: 5 Cocker Spaniel: 6 Mix breed: 9			

**Note:** Continuous variables are expressed as mean, standard deviation (SD), coefficient of variation (CV), and range. Age is expressed in years, weight in kilograms (kg), height in centimetres (cm), and BMI in kg/m^2^. For breed distribution, numbers indicate the number of dogs represented in each breed category.

**Table 2:** Thickness values of the SIT measured in the proximal and distal regions of the left and right tendons.

	Mean (mm)	SD (mm)	Coefficient of Variation	Range (mm)
**Right proximal tendon region**	2.02	0.46	0.23	2.23
**Right distal tendon region**	6.78	1.75	0.26	6.44
**Left proximal tendon region**	1.91	0.49	0.26	1.92
**Left distal tendon region**	6.13	1.61	0.26	6.2

**Note:** Continuous variables are expressed in millimetres (mm) as mean, standard deviation (SD), coefficient of variation (CV), and range. Sample size for each measurement was *n* = 35.

**Table 3:** Mean ultrasonographic echogenicity values of the canine SIT measured in the proximal and distal regions of the left and right tendons, as well as in the trapezius muscle.

	Mean (Grey)	SD (Grey)	Coefficient of Variation	Range (Grey)
**Right SIT proximal region**	71.26	21.10	0.29	81.62
**Left SIT proximal region**	69.90	20.63	0.29	89.54
**Right SIT distal region**	19.99	9.62	0.48	35.29
**Left SIT distal region**	30.62	16.36	0.53	62.43
**Right trapezius muscle**	11.80	8.54	0.72	34.13
**Left trapezius muscle**	11.35	12.40	1.09	56.79

**Note:** Echogenicity values are expressed as ultrasound grayscale intensity values. Continuous variables are presented as mean, standard deviation (SD), coefficient of variation (CV), and range. Sample size for each measurement was *n* = 35.

**Table 4:** Corrected relative ultrasonographic echogenicity values of the canine SIT measured in the proximal and distal regions of the left and right tendons.

	Mean (Grey)	SD (Grey)	Coefficient of Variation	Range (Grey)
**Right SIT proximal region**	59.56	16.24	0.27	60.63
**Left SIT proximal region**	58.55	16.06	0.27	69.97
**Right SIT distal region**	18.19	9.27	0.51	42.38
**Left SIT distal region**	19.26	11.89	0.62	46.41

**Note:** Echogenicity values were normalised using the trapezius muscle as reference tissue and are expressed as ultrasound grayscale intensity values. Continuous variables are presented as mean, standard deviation (SD), coefficient of variation (CV), and range. Sample size for each measurement was *n* = 35.

**Table 5:** Pearson correlation analysis between biological parameters and ultrasound-derived SIT measurements, including proximal and distal tendon thickness and corrected relative echogenicity values.

Variable 1	Variable 2	Pearson r	95% CI Lower	95% CI Upper	Raw *p*-Value	Holm-Adjusted *p*-Value
**Mean tendon thickness distal region**	**Age**	−0.002	−0.356	0.353	0.9919	1
	**Weight**	0.72	0.491	0.856	<0.0001	0.0016
	**Height**	0.665	0.406	0.825	<0.0001	0.0016
	**BMI**	−0.03	−0.38	0.328	0.8722	1
**Mean tendon thickness proximal region**	**Age**	0.041	−0.259	0.334	0.7899	1
	**Weight**	0.47	0.202	0.673	0.0013	0.0182
	**Height**	0.47	0.202	0.673	0.0013	0.0182
	**BMI**	−0.055	−0.346	0.246	0.7216	1
**Mean tendon echogenicity distal region**	**Age**	0.164	−0.19	0.48	0.3614	1
	**Weight**	0.157	−0.197	0.474	0.3844	1
	**Height**	0.271	−0.08	0.562	0.1268	1
	**BMI**	−0.135	−0.457	0.218	0.4525	1
**Mean tendon echogenicity proximal region**	**Age**	−0.157	−0.475	0.197	0.3829	1
	**Weight**	0.199	−0.155	0.507	0.268	1
	**Height**	0.122	−0.231	0.447	0.4975	1
	**BMI**	0.071	−0.279	0.405	0.6938	1

**Note:** Biological parameters include age, weight, height, and body mass index (BMI). SIT measurements include proximal and distal tendon thickness (expressed in millimetres) and corrected relative echogenicity values (expressed as ultrasound grayscale intensity values). Correlation coefficients (r), 95% confidence intervals (CI), and corresponding *p*-values are presented. *p*-values were adjusted for multiple comparisons using the Holm–Bonferroni method. Statistical significance was defined as adjusted *p* < 0.05.

## Data Availability

The data supporting the findings of this study are available from the corresponding author upon reasonable request.

## Abbreviations

The following abbreviations are used in this manuscript:

CT	Computed tomography
BMI	Body mass index
MRI	Magnetic resonance imaging
ROI	Region of interest
SD	Standard deviation
SIT	Supraspinatus insertion tendon
